# Selective Modulation of Osteoclast Function by *Bothrops moojeni* Venom and Its Fractions: Implications for Therapeutic Targeting in Bone Diseases

**DOI:** 10.3390/toxins17030141

**Published:** 2025-03-15

**Authors:** Fernanda D’Amélio, Hugo Vigerelli, Isabel de Fátima Correia Batista, Rodrigo Pinheiro Araldi, Álvaro R. B. Prieto-da-Silva, Daniel Carvalho Pimenta, Irina Kerkis

**Affiliations:** 1 Laboratory of Genetics, Butantan Institute, São Paulo 05503-900, Brazil; f.damelio@fundacaobutantan.org.br (F.D.); rodrigo.pinheiro.araldi@gmail.com (R.P.A.); alvaro.prieto@butantan.gov.br (Á.R.B.P.-d.-S.); 2 The Postgraduate Program in Toxinology, Butantan Institute, São Paulo 05503-900, Brazil; 3 Centre of Excellence in New Target Discovery (CENTD), Butantan Institute, São Paulo 05503-900, Brazil; hugo.barros@butantan.gov.br (H.V.); isabel.batista@butantan.gov.br (I.d.F.C.B.); 4 Laboratory of Biochemistry and Biophysics, Butantan Institute, São Paulo 05503-900, Brazil; dcpimenta@butantan.gov.br

**Keywords:** *Bothrops moojeni*, high- and low-molecular-weight fraction, osteoclast, morphology, function, secretome, molecular targets

## Abstract

Our study explores the differential effects of *Bothrops moojeni* venom and its fractions on osteoclast (OC) morphology, function, and osteoclastogenesis. The crude venom and its high-molecular-weight (HMW) fraction disrupt critical OC processes, including F-actin ring formation and mitochondrial distribution, thereby impairing bone resorption. These components primarily target cytoskeletal integrity and transcription regulation, with the OBSCN gene playing a direct role in OC function. In contrast, the low-molecular-weight (LMW) fraction selectively modulates OCs without significant cytoskeletal alterations. It influences vital cellular signaling pathways, notably through FNIP1 and FNIP2, essential for OC differentiation and function. This suggests a more targeted therapeutic approach with potentially fewer off-target effects. The venom also alters cytokine production, increasing IL-6 and IL-10 levels. Elevated IL-6 levels promote osteoclastogenesis and bone resorption, while IL-10 appears to counterbalance these effects through a regulatory feedback mechanism. Secretome analysis reveals that the crude venom and HMW fraction disrupt proteins involved in membrane trafficking and structural integrity. In contrast, the LMW fraction influences matrix remodeling, energy metabolism, and gene regulation. Gene interaction analysis LMW fraction post-treatment identifies FNIP1 and FNIP2 as critical targets involved in osteoclastogenesis. The observed changes in gene expression, including those related to immune response, energy metabolism, and chromatin remodeling, provide insights into the venom’s impact on bone health. Overall, the LMW fraction shows promise for drug development due to its selective implications and potential for fewer side effects, offering a more precise approach to treating bone diseases.

## 1. Introduction

*Bothrops moojeni*, commonly known as the Brazilian Lancehead or Caatinga pit viper, is a large and highly venomous snake native to South America’s Cerrado and Caatinga regions. Its venom is a potent, complex mixture of bioactive molecules, including metalloproteinases, phospholipases A2, and neurotoxins, contributing to its effects on blood coagulation, cell membranes, and tissue integrity. It is known for inducing hemorrhage, thrombosis, and extensive tissue damage through cytotoxic and myotoxic activities, and also disrupts nerve transmission [1,2,3,4]. The venom’s components also holds promise for therapeutic research, with potential applications in the treatment of blood disorders, cancer, and pain management [5,6].

Osteoclasts (OCs) are specialized multinucleated cells essential for bone matrix resorption, systemic calcium regulation, and skeletal remodeling. Originating from the monocyte/macrophage lineage, OCs undergo a meticulously regulated differentiation process orchestrated by cytokines, growth factors, and hormones. This intricate network of signaling pathways maintains the balance between bone resorption and formation, which is crucial for skeletal integrity and mineral homeostasis throughout life [7,8].

In the context of bone diseases, mature and polarized OCs represent critical targets for therapeutic intervention. Recent research highlights the significance of focusing on the maturation phase of OCs, where specific signaling pathways and metabolic alterations play pivotal roles [9,10]. Targeting these late-stage processes offers a refined strategy to inhibit excessive bone resorption while preserving the delicate equilibrium with bone formation, thereby providing a promising approach for the more effective treatment of conditions such as osteoporosis [11].

Primary osteoclast (OC) cultures are established from human bone marrow mononuclear cells or peripheral blood monocytes, which are then induced to differentiate by exposure to macrophage colony-stimulating factor (M-CSF) and the receptor activator of nuclear factor kappa-B ligand (RANKL) [12]. Culturing OCs from mixed peripheral blood cells provides a model that closely mirrors the in vivo environment, where OCs interact with a diverse array of immune cells. These interactions, mediated through cytokines and signaling molecules, significantly influence OC differentiation and function [13,14,15,16].

This culture system is a powerful tool for elucidating the mechanisms of osteoclastogenesis, performing bone resorption assays, and assessing the impact of pharmacological or genetic interventions on OC activity. Its relevance in reflecting the complex interplay between OCs and their microenvironment makes it indispensable for advancing our understanding of bone biology and developing targeted therapies.

This study aims to investigate the differential effects of *B. moojeni* venom, including its high (HMW)- and low-molecular-weight (LMW) fractions, on OC morphology, function, and resorptive activity. It explores how these venom components alter key cellular processes, such as F-actin ring formation, mitochondrial distribution, and cytokine production. Additionally, the study seeks to uncover venom-induced gene expression changes and identify molecular targets involved in osteoclastogenesis, with the goal of highlighting potential therapeutic strategies for treating bone diseases.

## 2. Results

### 2.1. Effect of B. moojeni Crude Venom and Its Fractions on Osteoclast Morphology and Reabsorption Function: F-Actin Ring Integrity and Mitochondrial Distribution

Our group has previously characterized OC morphology, performed Tartrate-Resistant Acid Phosphatase (TRAP) staining, and selected non-toxic concentrations of *B. moojeni* venom and its fractions [17]. Building on this foundation, we employed confocal microscopy in the current study to conduct a more detailed analysis of cell morphology and assess the integrity of F-actin rings—structures critical for the OC resorption process. These rings are essential for the initial sealing and synthesis of components such as cathepsin K, MMP9, and TRAP, which are integral to the degradation of the mineralized bone matrix. The disruption or rupture of these cellular structures can lead to compromised resorption efficiency and altered quality, as incomplete sealing affects the process [18].

**Figure 1:**
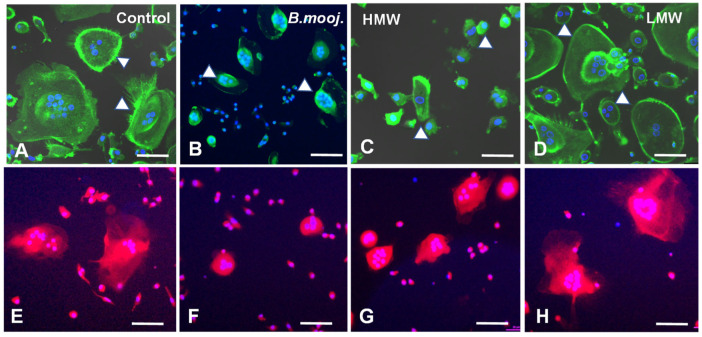
Effects of *Bothrops moojeni* venom and its fractions on OC morphology, F-actin Ring integrity, and mitochondrial intracellular distribution. Day 15 of OC differentiation. (**A**) A representative image of the control group, displaying intact F-actin rings of normal sizes with cytoplasmic projections. (**B**) OCs treated with *B. moojeni* venom [5 µg/mL] exhibit reduced cytoplasm and disrupted F-actin rings. (**C**) The group treated with HMW fraction [5 µg/mL] shows OCs with reduced cytoplasm and compromised F-actin rings. (**D**) The LMW fraction-treated group [1 µg/mL] presents OCs similar in size and morphology to the control group. F-actin is stained with Phalloidin (green), and nuclei are stained with DAPI (blue). (**E**) In the control group, mitochondria are dispersed throughout the cytoplasm and are found near the nucleus. (**F**) OCs treated with *B. moojeni* venom [5 µg/mL] show mitochondria concentrated near the nucleus and reduced cytoplasm. (**G**) The HMM-treated group [5 µg/mL] also shows mitochondria close to the nucleus with reduced cytoplasm. (**H**) In the LMM-treated group [1 µg/mL], mitochondria are dispersed and located near the nucleus. Mitochondria are stained red, and nuclei are stained with DAPI (blue). Scale bar: (**A**–**H**) = 100 µm.

Phalloidin is a highly selective bicyclic peptide used for staining F-actin filaments. Phalloidin staining revealed that, on day 15, untreated OCs cultured on a plastic surface exhibited well-formed, intact F-actin rings with extensive cytoplasmic projections (Figure 1A). In stark contrast, OCs exposed to crude *B. moojeni* venom [5 µg/mL] and its HMW fraction [5 µg/mL] show a marked reduction in size and significant disruptions in F-actin ring integrity, accompanied by a noticeable decrease in cytoplasmic volume (Figure 1B,C). OCs treated with the LMW fraction [1 µg/mL] maintain a size comparable to controls but display thinner F-actin rings and fewer cytoplasmic projections (Figure 1D). These structural alterations compromise the resorptive function of the OC membrane.

Mitochondria are crucial for OC absorption, primarily play a role by generating ATP to power cytoskeletal movement. The cellular location of mitochondria reflects the OCs’ functional status; when mitochondria are closer to the nucleus, absorptive activity is reduced, whereas mitochondria located further from the nucleus are associated with higher absorptive activity [19,20]. In the positive control with MitoTracker labeling (Figure 1E), mitochondria in OCs are observed near the nucleus and dispersed throughout the cytoplasm. In contrast, OCs treated with crude venom and the HMW fraction predominantly display mitochondria closer to the nucleus, indicating impaired absorptive function (Figure 1F,G). The mitochondrial distribution in the LMW-treated groups resembles that of the control group (Figure 1E).

### 2.2. Impact of B. moojeni Venom and Its Fractions on OC Absorptive Function: Inhibition of Bone Resorption and Associated Cellular Mechanisms

The absorption assay is pivotal in evaluating the impact of venom on OC functionality, specifically when assessing whether morphological changes and other functional parameters indicated a decrease or increase in activity after OC exposure to a mineralized matrix. This assay measures the absorptive capacity of OCs on a mineralized surface, simulating the bone matrix and serving as a functional indicator of cellular activation. This approach allowed us to assess the influence of venom on the overall process of bone resorption.

**Figure 2:**
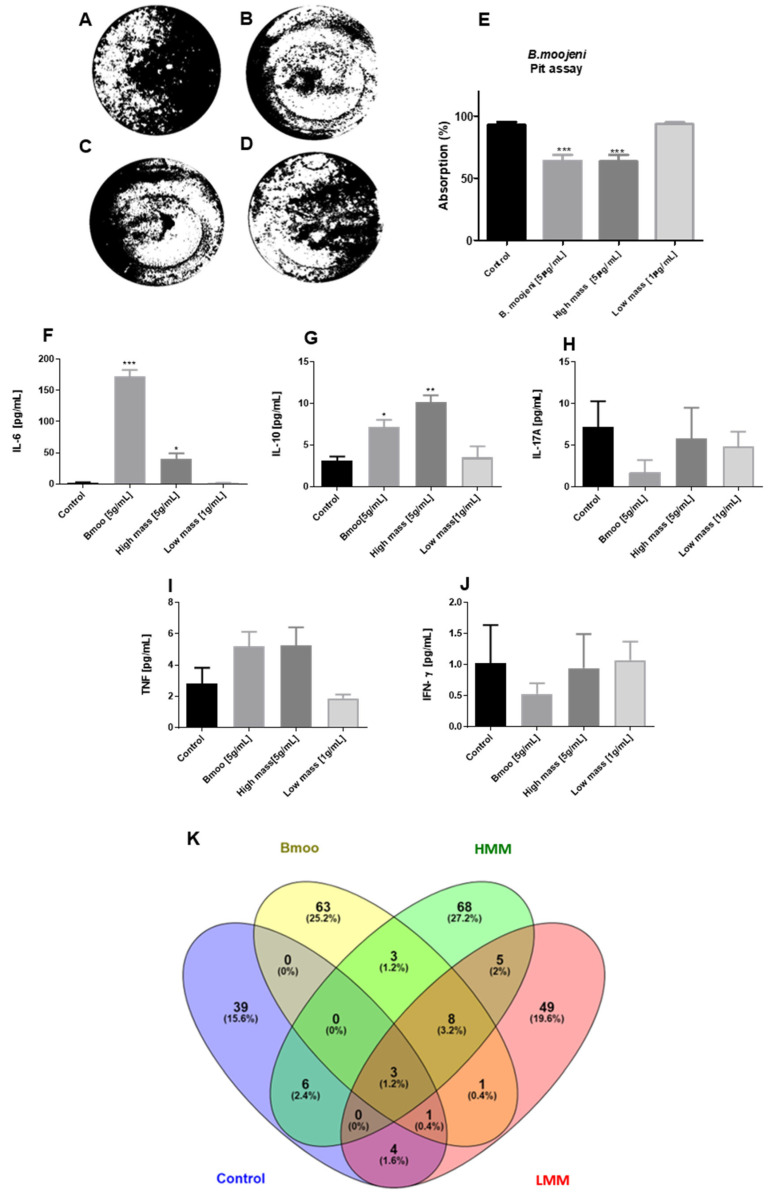
Effect of *B. moojeni* crude venom and its fractions on OC resorptive activity and its quantification (**A**–**E**). Day 15 of OC differentiation. (**A**) A representative image of the OC positive control group, showing typical resorption pits. (**B**) OCs treated with *B. moojeni* venom [5 µg/mL] exhibit decreased resorptive activity, as indicated by fewer resorption pits. (**C**) A similar reduction in resorption activity is observed in OCs treated with the HMW fraction. (**D**) OCs treated with the LMW fraction demonstrate resorption activity comparable to the positive control. Silver nitrate staining was employed to visualize resorbed areas (white), while non-resorbed areas appear black. (**E**) The bar graph quantifies the resorption activity of OCs under different treatment conditions. Both the *B. moojeni* venom [5 µg/mL] and the HMW fraction-treated groups show a significant decrease in resorptive activity compared to the control group. Conversely, the LMW fraction-treated group [1 µg/mL] exhibits resorption activity like that of the control. Data analysis was performed using GraphPad Prism version 8.0, with statistical significance determined at *p* < 0.01. (**F**–**J**) Cytokine secretion profiles following osteoclast treatment with *Bothrops moojeni* crude venom and its fractions. Cytokine levels were measured on day 15 of OC differentiation following treatment with *B. moojeni* crude venom and its fractions. The panels represent the secretion levels of various cytokines: (**A**) IL-6, (**B**) IL-10, (**C**) IL-17A, (**D**) TNF-α, and (**E**) IFN-γ. These data provide insights into the inflammatory and regulatory responses of OCs upon exposure to venom components. (**K**) Veen plot with the percentages of similar and unique proteins compared to the study groups. Statistics were performed using GraphPad Prism version 8.0.1, One-way ANOVA. *p*-values: * *p* < 0.05 vs. control group, ** *p* < 0.01 vs. control group, *** *p* < 0.001 vs. control group.

The visual representation of the absorption plate delineates areas resorbed by OCs (black) versus non-resorbed fields (white), with quantification performed via specialized software Image J 1.54 (Figure 2A–E). Results from the assay indicated that both the crude venom and the HMW fraction significantly inhibited OC absorption when compared to the control group (Figure 2A–C). In contrast, treatment with the LMW fraction did not demonstrate a strong inhibitory effect like that of the positive control (Figure 2A,D). Quantitative analysis revealed that OCs treated with *B. moojeni* crude venom and its HMW fraction [5 µg/mL] exhibited a significant reduction in resorptive activity relative to the control group (Figure 2E). Conversely, the LMW-treated group showed no significant difference from the control, indicating neither a decrease nor an increase in absorption (Figure 2E).

### 2.3. Distinct Cytokine Modulation in Osteoclastogenesis by B. moojeni Venom and Its Fractions: Insights into Pro-Inflammatory and Regulatory Responses

We evaluated the secretion of five cytokines directly associated with osteoclastogenesis and inflammation. IL-17A and TNF-α are known to promote osteoclastogenesis by upregulating RANKL expression while downregulating OPG, thereby intensifying bone resorption. Conversely, IFN-γ can inhibit osteoclastogenesis by disrupting RANKL signaling and enhancing OPG expression. IL-6 performs a pro-inflammatory role, facilitating OC differentiation, whereas IL-10 functions as an anti-inflammatory cytokine, potentially inhibiting bone resorption through the suppression of OC formation and activity [21,22].

We treated OCs with crude venom from *B. moojeni* and its HMW and LMW fractions. On day 15, supernatants were collected to assess cytokine secretion. Notably, regarding OCs treated with *B. moojeni* venom, those treated with the HMW fraction exhibited significantly elevated levels of IL-6 and IL-10 compared to the positive control. In contrast, the LMW fraction did not elicit a significant cytokine response (Figure 2F,G). Additionally, quantitative analysis revealed low levels of IL-17A, TNF, and IFN, with no statistically significant differences observed between the control and treated groups (Figure 2H–J). These findings suggest that the venom components do not significantly affect late-stage osteoclastogenesis, as evidenced by the unchanged levels of IL-17A, TNF-α, and IFN-γ. This is consistent with the understanding that RANKL expression, which is crucial for OC differentiation, is primarily associated with the early stages of OC development rather than with mature, polarized osteoclasts.

However, the elevated IL-6 and IL-10 levels seen in response to the crude venom and its HMW fraction indicate the complex modulation of cytokine activity, potentially influencing OC function through pro-inflammatory and immunoregulatory pathways.

Characterization of Secreted Proteins from Osteoclasts Grown on Mineralized Substrate: Impact of *B. moojeni* Venom and Its Fractions on Molecular Function and Pathways.

Mature and polarized OCs proteins, secreted on day 15 of differentiation, were precisely identified by comparing mass spectrometry and gas chromatography results against a human database (IDPROT) (Supplementary Materials Table S1). The analysis revealed distinct protein profiles for treatments with crude venom, high-molecular-weight fractions, and low-molecular-weight fractions. Crude venom treatment highlighted proteins such as Zinc Finger Protein (GLIS2), Desert Hedgehog Protein (DHH), and Nucleotide-binding Oligomerization Domain-containing Protein 1 (COL7A1), affecting transcription, signaling, and structural functions. In contrast, the high-molecular-weight fraction focused on Unconventional Myosin-Ia, Unconventional Myosin-Ic, Neural Cell Adhesion Molecule 2, Cadherin EGF LAG Seven-pass G-Type Receptor 2 (MYO1A, MYO1C, NCAM2, CELSR2), and Transforming Growth Factor Beta-3 Proprotein (TGFB3), impacting cellular movement, cell interactions, and bone metabolism. The low-molecular-weight fraction emphasized proteins like Alpha-1-antitrypsin, Creatine Kinase M-type, Lysine-specific Demethylase 6A, Phosphatidylinositol 3,4,5-trisphosphate 3-phosphatase, Vascular Endothelial Growth Factor Receptor 1 (SERPINA1, CKM, KDM6A, PTEN, FLT1), and fibrillin 1 (FBN1), which are involved in matrix remodeling, energy metabolism, and gene regulation. Comparatively, the positive control features Albumin, Pregnancy Zone Protein, Collagen Alpha-5(VI) Chain, Mitogen-Activated Protein Kinase 14 (ALB, PZP, COL6A5), and Tyrosine-Protein Kinase ITK (MAPK14), crucial for blood transport, matrix structure, and cellular signaling.

The enriched analysis (Venn diagram) reveals distinct and overlapping secretory profiles of OCs under various treatments. The crude venom treatment from *Bothrops moojeni* has the most extensive impact on the OC protein profile, as indicated by its possession of the largest number of unique proteins (63) and significant overlap with both the LMW and HMW fractions. This suggests that crude venom profoundly affects OC protein secretion, interacting with both LMW and HMW fractions. Conversely, the LMW and HMW fractions each exhibit distinct profiles with unique proteins, but also show notable interactions with the crude venom. Specifically, the LMW fraction has fewer unique proteins but considerable overlap with the venom, while the HMW fraction features a larger number of unique proteins and significant overlap with both the control and venom treatments. The Veen graph (Figure 2K) presents 39 proteins unique to the control, namely, 6 in common with the group treated with HMW, 4 in common with LMW, 1 in common with the group treated with crude venom and LMW, and 3 in common with crude venom, HMW, and LMW. The group treated with crude venom has 63 unique proteins, including 3 in common with HMW, 8 in common with HMW and LMW, 1 in common with LMW, 1 in common with control and LMW, and 3 in common with control, HMW, and low-mass variants. The group treated with HMW has 68 unique proteins, with 3 in common with raw venom, 8 in common with LMW and raw venom, 6 in common with control, and 3 in common with crude venom, LMW, and control. The group treated with LMW presents 49 unique proteins, with 5 in common with HMW, 1 in common with crude venom, 4 in common with control, 1 in common with crude venom and control, 8 in common with HMW and crude venom, and 3 in common with control, crude venom, and the HMW fraction.

### 2.4. Impact of B. moojeni Venom and Its Fractions on Osteoclast Secretome: Cellular and Molecular Pathways of Disruption and Adaptation

We analyzed proteins secreted by mature and polarized OCs, differentiated on a mineralized plate by day 15, and used mass spectrometry to evaluate how *B. moojeni* venom, including its HMW and LMW fractions, modulated or modified secreted proteins during its active functioning, uncovering the venom’s pathways of action. The mass data were enriched using Web Gestalt, with a focus on molecular function, biological process, cellular components, and pathways.

**Figure 3:**
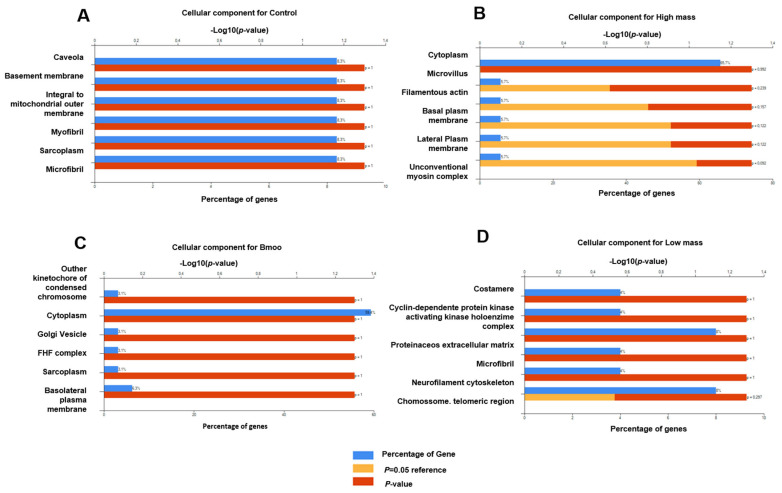
Enrichment analysis of cellular components. (**A**) Positive control group with only OCs without treatment; (**B**) study group OCs treated with HMW fraction; (**C**) group of OCs treated with crude venom; (**D**) group of OCs treated with LMW fraction.

Cellular components: Mass spectrometry analysis of OC proteins revealed critical components linked to caveolae, basement membrane, mitochondria, and muscle-related structures, underscoring the roles of membrane trafficking, extracellular matrix adhesion, energy metabolism, and contractile activity in bone resorption (Figure 3A). Treatment with *B. moojeni* venom perturbed proteins associated with the kinetochore, cytoplasmic and Golgi vesicles, and the FHF complex, suggesting potential disruptions in cell cycle regulation, intracellular trafficking, and ion homeostasis, alongside alterations in cytoskeletal integrity and cellular polarization (Figure 3B). Exposure to the HMW fraction resulted in significant disruptions to proteins involved in cytoplasm, microvilli, filamentous actin, and plasma membranes, indicating compromised cytoskeletal integrity and cell polarity (Figure 3C). Analysis of the LMW fraction implicated costamere and CDK holoenzyme proteins in stability and cell cycle regulation, with further potential effects on extracellular matrix interactions, neurofilaments, and chromosomal stability, despite statistical insignificance (Figure 3D).

**Figure 4:**
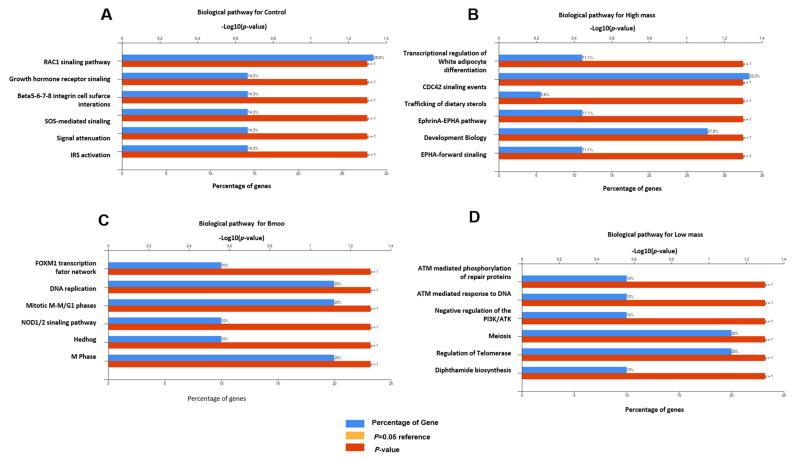
Enrichment analysis of molecular function. (**A**) Positive control group with only OCs without treatment; (**B**) study group OCs treated with HMW fraction; (**C**) group of OCs treated with crude venom; (**D**) group of OCs treated with the LMW fraction.

Molecular function: The positive control analysis of OC proteins revealed significant enrichment in categories crucial for matrix remodeling and signaling, including extracellular matrix components, GTPase activators, voltage-gated ion channels, GEFs, and protein tyrosine kinases, underscoring their essential roles in OC function on mineralized substrates (Figure 4A). In contrast, *B. moojeni* venom-treated OCs displayed notable alterations in protein functions, including transcription factor binding, structural modifications, metal ion binding, and the activation of apoptotic pathways, with disruptions in skeletal protein interactions and receptor-mediated signaling pathways (Figure 4B). The HMW fraction of the venom induced diverse effects, impacting motor function, apoptotic protease activity, DNA repair, transmembrane receptor PTK activity, lipoprotein receptor function, and serine/threonine kinase signaling, indicating broad disruptions in OC signaling and lipid metabolism (Figure 4C). The LMW fraction highlighted significant activities related to serine/threonine kinase signaling, transcriptional regulation, lipid phosphatase function, GEFs activity, and apoptotic protease activation, alongside extracellular matrix remodeling, suggesting complex impacts on OC signaling and matrix interactions (Figure 4D).

**Figure 5:**
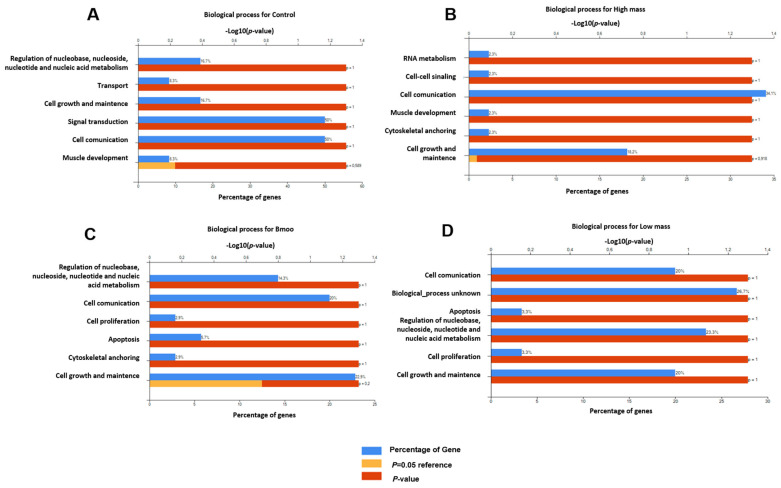
Enrichment analysis of biological processes. (**A**) Positive control group with only OCs without treatment; (**B**) study group OCs treated with HMW fraction; (**C**) group of OCs treated with crude venom; (**D**) group of OCs treated with the LMW fraction.

Biological processes: The positive control analysis of OC proteins demonstrated substantial enrichment in processes related to nucleobase, nucleotide, nucleoside, and nucleic acid metabolism, alongside transport, cell growth, maintenance, signal transduction, cell communication, and muscle development, highlighting the intricate regulatory mechanisms governing OC function and adaptation (Figure 5A). In contrast, OCs treated with *B. moojeni* venom showed significant enrichment in similar metabolic and growth-related processes, with additional impacts on cell communication, proliferation, apoptosis, and cytoskeletal anchoring, indicating broad disruptions to cellular function and structural integrity (Figure 5B). The HMW fraction of the venom notably affected RNA metabolism, cell–cell signaling, intercellular communication, muscle development, and cytoskeletal anchoring, suggesting profound influences on essential cellular mechanisms and structural dynamics (Figure 5C). The LMW fraction enriched processes related to cell communication, apoptosis, proliferation, growth maintenance, and nucleic acid metabolism, with some proteins linked to unknown biological processes, hinting at potential novel pathways requiring further exploration (Figure 5D).

**Figure 6:**
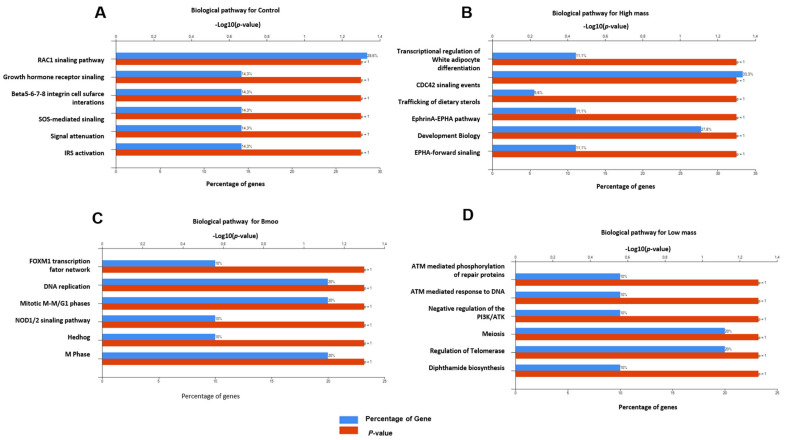
Enrichment analysis of biological pathway. (**A**) Positive control group with only OCs without treatment; (**B**) study group OCs treated with HMW fraction; (**C**) group of OCs treated with crude venom; (**D**) group of OCs treated with LMW fraction.

Biological pathway: The positive control analysis of OC proteins revealed enrichment in key signaling pathways, including RAC1 and growth hormone receptor signaling, as well as beta integrins (β5–β8), crucial for adhesion and signaling. SOS-mediated signaling, signal attenuation, and IRS activation were also noted, reflecting complex regulatory mechanisms essential for OC function (Figure 6A). In *B. moojeni*-treated OCs, the FoxM1 transcription factor network, mitotic M-M/G1 phases, NOD1/2 signaling, and Hedgehog pathways were enriched, indicating disruptions in transcriptional regulation, cell cycle, and immune responses (Figure 6B). The HMW fraction significantly impacted transcriptional regulation, adipocyte differentiation, CDC42 signaling, and sterol trafficking, suggesting effects on gene expression, lipid metabolism, and cellular interactions (Figure 6C). The LMW fraction enriched pathways related to DNA repair, PIK3/AKT network regulation, meiosis, telomerase activity, and depthamide biosynthesis, indicating potential disruptions in genomic stability and key signaling processes essential for OC function (Figure 6D).

### 2.5. Gene Expression Profiles in Osteoclast Differentiation: Insights into Immune and Osteogenic Interactions in Mixed Peripheral Blood Cell Cultures

**Figure 7:**
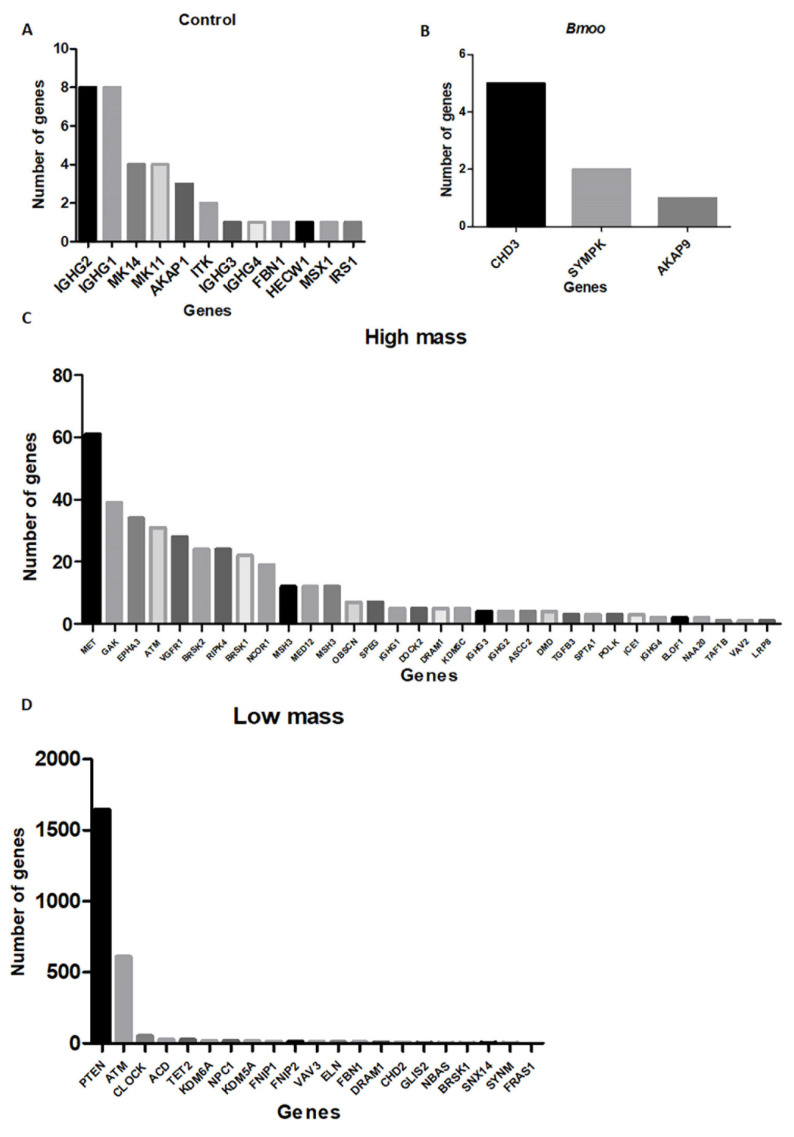
Graph with count of statistically relevant genes in number. (**A**) Positive control group with only OCs without treatment; (**B**) group treated with crude venom; (**C**) group treated with HMW fraction; (**D**) group treated with LMW fraction.

**Figure 8:**
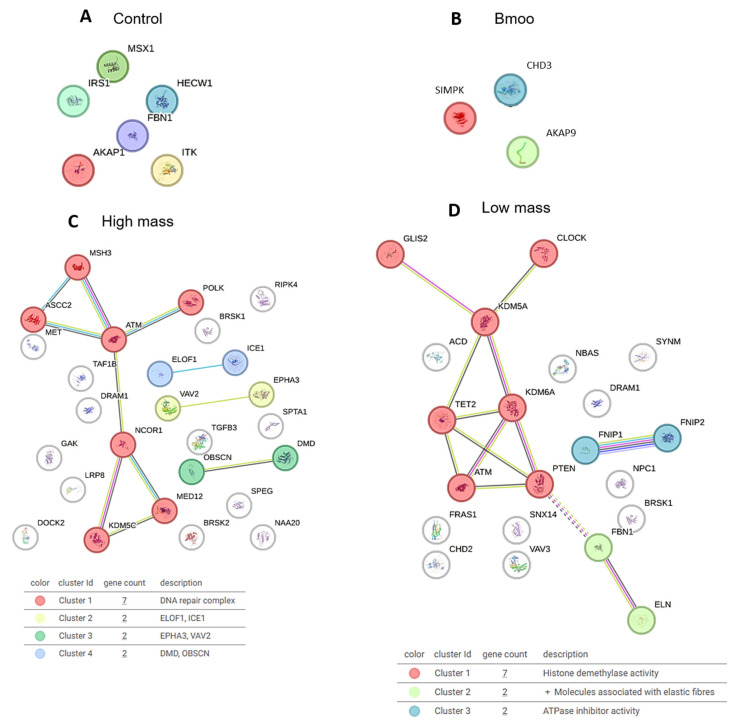
STRING analysis of gene interactions for control OCs (**A**) and OCs treated with *B. moojeni* crude venom (**B**) and its HMW (**C**) and LMW (**D**) fractions, along with gene enrichment analysis (adjusted *p*-values). (**A**,**B**) No clusters were identified for proteins in control culture and post-treatment with crude venom. (**C**) Four distinct clusters were observed: Cluster 1—DNA repair complex; Cluster 2—ELOF1 and ICE1; Cluster 3—EPAH3 and VAV2; and Cluster 4—DMD and OBSCN. (**D**) Three clusters were detected: Cluster 1—histone demethylase activity; Cluster 2—molecules associated with elastic fibers; and Cluster 3—ATPase inhibitor activity.

The gene list for control OCs (Table 1) includes immunoglobulin genes (e.g., immunoglobulin heavy chain gamma 1–4, IGHG2, IGHG1), kinases (e.g., mitogen-activated protein kinase, MK14, MK11), and other genes such as AKAP1 (A-kinase anchoring protein 1), FBN1 (fibrillin 1), HECW1 (HECT, C2, and WW domain-containing E3 ubiquitin protein ligase 1), MSX1 (Msh Homeobox 1), and IRS1 (insulin receptor substrate 1). Immunoglobulin genes suggest the occurrence of immune-related interactions due to the presence of B cells and other immune cells in the culture. MK14 and MK11 are critical for osteoblast differentiation, while AKAP1 is involved in mitochondrial function and apoptosis, relevant to both osteoblast and OC differentiation. ITK, a T-cell kinase, indicates potential immune influences on osteogenesis. FBN1 affects connective tissue and bone structure, and HECW1 is involved in protein degradation, with both being important for bone biology. MSX1 is linked to craniofacial development, and IRS1 is crucial for insulin signaling and osteoblast differentiation. These genes highlight the interplay between immune factors and OC function, reflecting the mixed cell population derived from peripheral blood and mimicking the bone marrow environment (Figure 7A). The STRING analysis did not reveal any significant interactions or cluster formations between the genes (Figure 8A).

### 2.6. Molecular Insights into Osteoclast Modulation by B. moojeni Venom and Its Fractions: Differential Expression and Functional Implications of Key Regulatory Genes

The differential expression of CHD3 (Chromodomain-helicase-DNA-binding protein 3), SYMPK (Symplekin), and AKAP9 (A-kinase anchor protein 9) genes in OCs treated with *B. moojeni* venom (Table 2) suggests that the venom influences crucial regulatory mechanisms within these cells (Figure 8C). CHD3 affects chromatin remodeling and gene regulation, potentially altering OC-specific gene expression. SYMPK influences mRNA stability and translation, influencing the production of proteins essential for OC formation. AKAP9 is involved in PKA signaling, which is vital for regulating OC differentiation and bone resorption. Changes in these genes’ expression can significantly influence osteoclastogenesis (Figure 7B). The STRING analysis did not identify any significant interactions or clusters among the genes (Figure 8B).

The thirty-three genes identified as statistically significant following the treatment of OCs with the HMW fraction (Supplementary Materials Table S1) of *Bothrops moojeni* venom play diverse and critical roles in osteoclastogenesis (Figure 8B). Genes directly involved in osteoclastogenesis include MET (Hepatocyte Growth Factor Receptor), a receptor tyrosine kinase that influences osteoclast (OC) differentiation and bone resorption through growth and survival signaling. EPHR3 (Eph Receptor B3) regulates OC differentiation and activity via interactions with ephrin ligands, affecting OC formation, survival, and bone resorption. MED12 (Mediator Complex Subunit 12) is a critical component of the mediator complex, which is a multi-protein assembly essential for regulating gene transcription. It can modulate the gene expression necessary for OC differentiation and function, affecting bone resorption and remodeling. MSH3 (MutS Homolog 3) can maintain DNA repair and genomic stability during OC differentiation, which is crucial for proper OC function and bone metabolism (Figure 7C).

Genes related to angiogenesis and bone remodeling include VEGFR1 (Vascular Endothelial Growth Factor Receptor 1), suggesting it may play a role in angiogenesis, such as interacting with and potentially influencing OC function and bone remodeling processes. Genes involved in transcriptional regulation and bone metabolism include TGFB3 (Transforming Growth Factor Beta 3) and NCOR1 (Nuclear Receptor Corepressor 1), which are key regulators of transcription and bone metabolism, influencing OC differentiation. Additionally, genes involved in cell migration, apoptosis, and cytoskeletal organization include DOCK2 (Dedicator of Cytokinesis 2), DRAM1 (DNA Damage-Regulated Autophagy Modulator 1), and SPTA1 (Spectrin Alpha, Erythrocytic 1), which play vital roles in modulating cell migration, apoptosis, and cytoskeletal organization, which are essential for OC function (Figure 7C).

Genes involved in cell cycle regulation, signaling, and stress responses, such as GAK (Cyclin G Associated Kinase) and BRSK1/2 (BR Serine/Threonine Kinase 2), may influence osteoclast proliferation and activity. ATM (ATM Serine/Threonine Kinase) and MSH3 are crucial for stress response and DNA repair, maintaining osteoclast genomic integrity. IGHG (Immunoglobulin Heavy Constant Gamma) may reflect immune system interactions, while KDM5C (Lysine Demethylase 5C) and POLK (DNA Polymerase Kappa) are involved in gene regulation and DNA repair, affecting OC behavior. The RIPK4 gene, a receptor-interacting protein kinase, is involved in signaling, stress responses, and immune interactions. Lastly, OBSCN (Obscurin) plays a role in cytoskeletal organization, muscle structure, and signaling pathways (Figure 7C).

SPEG (Striated Muscle Enriched Protein Kinase), ASCC2 (Activating Signal Cointegrator 1 Complex component), DMD (dystrophin), ICE1 (Interactor of Little Elongation Complex ELL Subunit 1), ELOF1 (Elongation Factor 1 Homolog), NAA20 (N-terminal acetyltransferase A subunit), TAF1B (TATA-box Binding Protein Associated Factor 1B), and LRP8 (Apolipoprotein E Receptor 2) do not directly influence osteoclastogenesis, but may affect it indirectly. SPEG is involved in muscle-specific kinase activity, ASCC2 in transcription regulation, DMD in structural support, ICE1 in transcription regulation, ELOF1 in protein synthesis, NAA20 in protein acetylation, TAF1B in transcription initiation, and LRP8 in lipid metabolism and signaling. VAV2 is indirectly connected to OC phenotypes. These genes illustrate the complex molecular network influencing OC biology and bone metabolism following treatment with the HMW fraction of *B. moojeni* venom (Figure 7C).

The STRING analysis of gene interactions post-treatment with the HMW fraction of *B. moojeni* venom revealed four distinct clusters. Among the clusters identified, Cluster 1 (ASCC2, MSH3, POLK, ATM, NCOR1, KDM5A, and MED12) is primarily involved in DNA repair and transcription regulation. While unrelated to osteoclastogenesis, genes like ATM and NCOR1 may influence it through their roles in stress responses and gene regulation. Cluster 2 (VAV2 and EPHA3) focuses on signaling and cellular communication, but VAV2 is not directly involved in osteoclastogenesis. Cluster 3 (OBSCN and DMD) deals with cytoskeletal organization and structural support, making OBSCN relevant to osteoclast function through its involvement in cytoskeletal dynamics. Cluster 4 (ELOF1 and ICE1) is related to protein synthesis and transcription regulation, with both genes potentially affecting osteoclastogenesis indirectly through their roles in cellular processes. Therefore, OBSCN is more directly linked to osteoclastogenesis (Figure 8C).

Following the treatment of OCs with the LMW fraction of *B. moojeni* venom (Supplementary Materials Table S2), twenty-one genes emerged as statistically significant, highlighting their involvement in crucial processes related to osteoclastogenesis and bone metabolism (Figure 8D). These findings suggest that these genes play regulatory roles in signaling pathways and have the potential to modulate OC function in response to LMW fraction exposure. KDM5A (Lysine Demethylase 5A) is directly involved in osteoclastogenesis. This gene affects the transcriptional landscape of OCs by modifying histones, thereby regulating the expression of genes essential for OC differentiation and function. Additionally, ELN (elastin) and FBN1 (fibrillin 1) are pivotal for maintaining an extracellular matrix structure, suggesting that alterations in these genes may influence bone remodeling by affecting the integrity of the matrix. The NSBA gene, also known as Nucleotide-Binding Oligomerization Domain Containing 1 (NOD1), is essential for regulating osteoclastogenesis, modulating inflammatory pathways that influence bone resorption. Alterations in the NSBA gene can impact osteoclastogenesis through their effects on signaling pathways that control OC differentiation and activity (Figure 7D).

VAV3 (Vav Guanine Nucleotide Exchange Factor 3) contributes to signaling pathways that could affect OC inflammation and apoptosis in the cell cycle, signaling, and stress responses. Similarly, FNIP1 and FNIP2 (Folliculin-Interacting Protein 1 and 2) are linked to cellular metabolism and signaling, potentially modulating OC activity through these processes. PTEN (Phosphatase and Tensin Homolog) and ATM (Ataxia Telangiectasia Mutated) play crucial roles in stress responses and the maintenance of genomic integrity, which could influence OC survival and function. CLOCK (Clock Circadian Regulator) and KDM6A (Lysine Demethylase 6A) are involved in circadian rhythms and epigenetic modifications, respectively. Their roles suggest they may regulate osteoclast differentiation through circadian and epigenetic mechanisms. TET2 (Tet Methylcytosine Dioxygenase 2) impacts transcriptional regulation, potentially affecting osteoclast function, while NPC1 (NPC Intracellular Cholesterol Transporter 1) is crucial for cholesterol and lipid transport, potentially influencing osteoclast activity. DRAM1 (DNA Damage-Regulated Autophagy Modulator 1) is associated with autophagy and apoptosis, linking its function to osteoclastogenesis through stress response mechanisms. CHD2 (Chromodomain Helicase DNA-binding protein 2), a chromatin remodeler, affects gene expression through chromatin modifications. An understanding of its role in osteoclastogenesis is emerging, with suggestions it may regulate gene expression programs necessary for OC differentiation and bone resorption (Figure 7D).

The FRAS1 gene, which stands for “Fraser Syndrome 1”, is crucial for extracellular matrix integrity and cell adhesion, alongside the SYNM gene that encodes a key cytoskeletal protein, contributes to cellular structural maintenance. These roles potentially influence osteoclastogenesis, the differentiation of bone-resorbing osteoclasts. The NBAS (Neuroblastoma Amplified Sequence) gene, essential for intracellular transport, and the SNX14 (Sorting Nexin 14) gene, involved in endosomal sorting, may impact osteoclastogenesis by modulating the trafficking of critical signaling molecules. NCAM2 (Neural Cell Adhesion Molecule 2), which is important for cell adhesion, and PZP (Pregnancy Zone Protein), which regulates immune responses and inflammation, could affect osteoclastogenesis through their roles in cellular interactions and inflammatory pathways. DPH5 (Diphthamide Biosynthesis 5), necessary for diphthamide biosynthesis and subsequent protein synthesis, might influence osteoclastogenesis by affecting the production of essential osteoclast proteins. The GLIS2 gene (Glis Family Zinc Finger 2) is a member of the GLIS family of transcription factors, which play essential roles in various biological processes and may play a role in bone metabolism and homeostasis. BRSK1 (Brk/Snk-related Kinase 1) has been previously described in OCs following HMW fraction pretreatment, highlighting its regulatory role in signaling pathways and suggesting its potential involvement in modulating OC function after exposure to venom components. Despite their known functions, the specific contributions of these genes to osteoclastogenesis are yet to be fully elucidated (Figure 7D).

The analysis of gene interactions identified three distinct clusters, each reflecting unique functional relationships. Additionally, several genes were found that did not fit into these clusters. Among the clusters identified, Cluster 3 (FNIP1 and FNIP2) is most directly related to osteoclastogenesis. This cluster focuses on cellular signaling and regulation, which are crucial for OC differentiation and function. FNIP1 and FNIP2 are involved in signaling networks that can affect OC activity. In contrast, Cluster 1 (GLIS2, CLOCK, KDM5A, TAT2, KDM6A, TET2, ATM, and PTEM) deals with transcriptional regulation and DNA damage response, while Cluster 2 (FBN1 and ELN) is associated with tissue elasticity and structural integrity. Although Cluster 1 may influence osteoclastogenesis indirectly through gene regulation, Cluster 3 is more directly linked to the signaling processes essential for OC function (Figure 8D).

## 3. Discussion

Our study demonstrates that *B. moojeni* venom and its fractions exert differential and profound effects on OC morphology, function, and osteoclastogenesis, revealing the intricate mechanisms underlying these disruptions. The crude venom and its HMW fraction significantly impair OC resorptive activity by disrupting F-actin ring formation and mitochondrial distribution, essential for cytoskeletal integrity and bone resorption [18,19,20]. This suggests that these venom components target critical structural and functional elements within OCs, substantially reducing their resorptive capabilities. In contrast, the LMW fraction exhibits a more selective impact, affecting OC function without significantly altering cytoskeletal structures or mitochondrial positioning. This indicates that the LMW fraction might interact with different molecular targets within OCs, leading to more nuanced modulation of their activity.

The venom’s ability to modulate cytokine production, as evidenced by elevated IL-6 and IL-10 levels, suggests the existence of a complex interplay between pro- and anti-inflammatory responses in OCs and their environment. The increase in IL-6, which promotes OC differentiation and bone resorption, indicates that venom components may enhance osteoclastogenic potential and create a pro-inflammatory environment conducive to bone degradation [21,22]. Conversely, the induction of IL-10 suggests a regulatory feedback mechanism that may temper the initial pro-inflammatory effects [23,24]. IL-10 is known to inhibit osteoclastogenesis by downregulating the production of osteoclastogenic cytokines such as TNF-α, IL-1, and IL-6 [21]. The lack of a similar cytokine response from the LMW fraction further emphasizes the role of molecular weight and structural complexity in determining the venom’s effects on OC function and cytokine modulation.

The analysis of OC-secreted proteins post-treatment and in control conditions highlights their distinct impacts on OC biology. Crude venom alters transcription and signaling with GLIS2 and DHH and modifies structural components via COL7A1 and ARHGAP24 [25,26,27,28]. The HMW fraction affects cellular movement and interactions through MYO1A, MYO1C, and NCAM2 while also influencing bone metabolism with TGFB3 [29,30,31,32]. The LMW fraction is notable for its role in matrix remodeling (SERPINA1), energy metabolism (CKM), and gene regulation (KDM6A, PTEN) [33,34,35,36]. In contrast, positive control proteins such as ALB and MAPK14 establish a baseline for assessing these treatments’ effects on OC functionality [37].

The enrichment secretome analysis following venom treatment reveals extensive alterations in OC signaling pathways, cellular components, and molecular functions. The crude venom and its fractions disrupt proteins involved in membrane trafficking, cytoskeletal integrity, and cellular polarization—critical OC function and bone resorption processes. The observed enrichment of transcription factor binding, metal ion binding, and apoptosis-related pathways in venom-treated OCs highlights venom’s broad regulatory impact, which can potentially lead to altered gene expression, cell stress, and apoptosis.

The identified genes in primary cultures of mature and polarized OCs secretome unveil complex mechanisms governing osteoclast function and regulation. The presence of immune factors such as IGHG2, IGHG1, IGHG3, and IGHG4 points to thepotential modulation of OC function and bone resorption by immune responses, suggesting significant interplay between immune factors and OC activity [25,26]. Kinases MK14 and MK11 are pivotal for OC differentiation and function through the p38 MAPK pathway, ensuring adequate OC maturation and activity [27,28]. AKAP1 plays a crucial role in supporting OCs’ energy needs and survival by maintaining mitochondrial function and regulating apoptosis, underscoring the importance of energy management in OC function [29,30].

The involvement of ITK indicates that T-cell signaling pathways may affect OC polarization and activity, revealing potential cross-talk between immune and OC signaling pathways [25,31]. FBN1 supports OC function and bone remodeling, highlighting the connection between matrix components and osteoclast activity. HECW1’s role in protein degradation further contributes to OC homeostasis by regulating protein turnover, which is vital for maintaining OC function [32]. MSX1’s influence on OC differentiation and bone remodeling integrates genetic regulation into OC development [33]. Finally, IRS1 links metabolic pathways to bone health, potentially affecting OC function through insulin signaling, connecting metabolic regulation with OC activity [34,35]. These genes reflect the multifaceted regulation of regular mature OCs and their critical role in bone health and disease.

The differential expression of CHD3, SYMPK, and AKAP9 in OCs post-treated with *B. moojeni* crude venom reveals critical insights into the venom’s impact on OC biology. CHD3, an ATP-dependent chromatin remodeler, is crucial for gene regulation and developmental processes [36,37]. While its specific role in osteoclastogenesis remains to be fully elucidated, its related protein, CHD9, is known to influence osteogenic differentiation [38]. SYMPK modulates mRNA stability and protein production and is vital for OC formation. Its role in polyadenylation and mRNA stability indicates that it may act as a key regulator of OC differentiation and activity by affecting gene expression and mRNA–microRNA interactions [39,40]. AKAP9, a Protein Kinase A (PKA) signaling pathway component, influences significantly OC differentiation and bone resorption. Despite limited research directly targeting AKAP9 in bone diseases, its function in anchoring PKA and regulating essential cellular processes underscores its potential as a therapeutic target in bone health [41].

The interplay of genes identified post-treatment with HMW venom fraction in OC biology orchestrates a complex network that governs vital aspects of OC differentiation, function, and survival, as well as their interactions with the bone matrix and immune system. Key regulators such as MET and EPHR3 drive osteoclastogenesis and bone resorption by influencing differentiation and activity [38]. MED12 integrates transcriptional regulation, essential for OC function, while MSH3 maintains genomic stability during OC differentiation, ensuring functional integrity homeostasis [39,40,41]. VEGFR1 modulates angiogenesis and bone remodeling, affecting OC function through its role in vascular dynamics. TGFB3 and NCOR1 further impact transcriptional regulation and bone metabolism, underscoring their roles in gene expression modulation [42,43]. Cell migration, apoptosis, and cytoskeletal organization are regulated by DOCK2, DRAM1, and SPTA1, which collectively influence OC functionality and turnover [42,43,44]. GAK and BRSK1/2 integrate cell cycle and signaling pathways crucial for OC proliferation and activity. Concurrently, ATM and MSH3 contribute to stress responses and DNA repair, preserving OC integrity [45,46]. IGHG reflects immune interactions and gene regulation, with implications for OC function through immune signaling. KDM5A and POLK underscore the importance of epigenetic modifications and DNA repair in regulating OC behavior [47,48].

The STRING analysis revealed four distinct protein clusters with key roles in OC biology. Cluster 1 is involved in DNA repair and transcriptional regulation, processes essential for genomic maintenance during osteoclastogenesis. Cluster 2 is focused on cell signaling, which is crucial for regulating OC activity in response to cytokines. Cluster 3, centered on cytoskeletal organization, includes OBSCN, a key regulator of cell adhesion, migration, and structural support, all of which are fundamental for bone resorption [49]. Cluster 4 is associated with protein synthesis, reflecting the high demand for the proteins required in OC function.

After treatment with the LMW fraction of *B. moojeni* venom, specific genes were identified as statistically significant due to changes in their expression profiles. This suggests that the venom components specifically target these genes, influencing their roles in crucial biological processes. The in-reach analysis further underscored these changes, highlighting their potential involvement in osteoclastogenesis and bone metabolism. Among these genes, KDM5A regulates gene expression in osteoclastogenesis by modifying histones, which affects the transcriptional landscape of OC differentiation [47]. Consequently, changes in KDM5A activity can affect OC function and bone resorption, affecting bone density and health. Elastin (ELN), secreted by elastogenic cells, forms fibers that contribute to the extracellular matrix (ECM) and bone remodeling. Although primarily associated with connective tissues, elastin influences OC function by interacting with the ECM, potentially affecting bone resorption processes [48]. Fibrillin 1 (FBN1), a key ECM component, affects bone metabolism by modulating TGFβ signaling. Deficiencies or defects in FBN1, as seen in Marfan syndrome, disrupt TGFβ activity and consequently affect OC differentiation and function. This underscores FBN1’s role in maintaining regular OC activity and bone homeostasis, highlighting its relevance in bone degeneration and related pathologies [49]. In turn, Vav3, a key guanine nucleotide exchange factor (GEF) in OCs, is critical for their function. Mice lacking Vav3 exhibit increased bone mass due to impaired OC function, including defective cell spreading, polarization, and resorption due to disrupted actin ring formation. Vav3 is essential for signaling downstream of αvβ3 integrin, and reintroducing it restores normal OC function, marking it as a potential therapeutic target [50,51]. FNIP1 and FNIP2 are crucial for regulating cellular metabolism and energy balance through the AMPK/mTOR pathway, which is vital for osteogenesis. They influence AMPK/mTORC1 localization and mitochondrial metabolism, linking them indirectly to osteoblast differentiation and bone formation. Their roles in energy homeostasis highlight their importance in osteogenic processes [48,50]. PTEN and ATM could be targeted by LMW to modulate OC survival and function by influencing stress responses and genomic integrity [36]. The changes in CLOCK and KDM6A can potentially affect OC differentiation and bone metabolism through circadian regulation and epigenetic modifications. The dysregulation of KDM6A can impair OC activity and contribute to bone diseases [50,51,52]. After treatment with the LMW fraction, the expression of TET2 and NPC1 was notably altered. TET2 plays a key role in regulating transcription through DNA methylation, which can affect OC differentiation and function. NPC1 is crucial for cholesterol and lipid transport, affecting OC activity and bone metabolism. The changes observed in these genes suggest that the venom components influence their roles in osteoclastogenesis and bone health [45]. DRAM1 was significantly altered after treatment, highlighting its role in regulating autophagy, which is crucial for OC differentiation and function. Proper DRAM1 activity supports OC maturation and bone resorption, essential for bone homeostasis. The dysregulation of DRAM1 can impair autophagy, leading to compromised OC function and bone loss, making it a promising therapeutic target for improving bone health [52,53]. Additionally, alterations in CHD2 were observed, highlighting the crucial role it plays in osteoclastogenesis by regulating genes essential for OC differentiation and activity. Its impact on chromatin dynamics suggests that CHD2 influences the transcriptional programs driving osteoclastogenesis and bone resorption, underscoring its potential as a critical regulator of OC function [54,55]. The changes observed in these genes suggest that the venom components influence their roles in osteoclastogenesis and bone health.

Gene interaction analysis highlights that Cluster 3, featuring FNIP1 and FNIP2, plays a pivotal role in osteoclastogenesis by influencing critical cellular signaling pathways [53,54,55]. These include integrin signaling and Rho GTPase pathways, vital for OC differentiation, adhesion, and bone resorption [56].

## 4. Conclusions

Our study reveals that *B. moojeni* venom and its HMW and LMW fractions profoundly influence OC morphology, function, and osteoclastogenesis. The crude venom and HMW fraction disrupt critical processes like F-actin ring formation and mitochondrial distribution, thereby impairing bone resorption. In contrast, the LMW fraction selectively modulates OC function without causing significant cytoskeletal alterations. This fraction targets key cellular signaling pathways involving FNIP1 and FNIP2, which are essential for OC differentiation and function, offering a more targeted approach with fewer off-target effects. Additionally, the venom alters cytokine production, elevating IL-6 and IL-10, highlightingthe complex interplay between pro- and anti-inflammatory responses. Secretome analysis reveals extensive changes in OC signaling pathways, cellular components, and gene expression. These insights underscore the LMW fraction’s potential for drug development, providing a precise and potentially safer approach to treating bone diseases.

## 5. Methodology

### 5.1. hPBMCs and OC Differentiation Protocol

Peripheral blood mononuclear cells (PBMCs) were isolated using Ficoll–Paque density gradient centrifugation (density 1.077 g/mL, Sigma-Aldrich^®^, St. Louis, MO, USA). Blood samples (20 mL) from healthy male volunteers (aged 25–40) were collected via venipuncture at the cubital fossa (Plataforma Brasil/CEP 1,806,596). The samples were diluted 1:1 with 0.9% saline and carefully layered onto Ficoll–Paque at a 1:3 ratio in conical tubes. Centrifugation was performed at 400× *g* for 20 min without braking, after which PBMCs were carefully collected. The cells were washed twice with saline and resuspended in 1 mL of differentiation medium, composed of α-MEM (Thermo Fisher Scientific, Waltham, MA, USA). It was adjusted to pH 7.4 and supplemented with 10% fetal bovine serum (LGC Biotecnologia, Granja Viana, Cotia, SP, Brazil), 25 ng/mL human M-CSF, 50 ng/mL human RANKL, 5 ng/mL human TGF-β1 (R&D Systems, Minneapolis, MN, USA), and 1 μM dexamethasone (Sigma-Aldrich^®^, St. Louis, MO, USA).

For OC differentiation assays, 6 × 10^5^ PBMCs were seeded into each 1.9 cm^2^ well and cultured in 200 μL of differentiation medium. The medium was refreshed twice weekly, with 50% of the volume replaced, for a total of 15 days.

### 5.2. F-Actin

To assess F-actin ring formation, a hallmark of OCs, cells were stained using Phalloidin conjugated to Alexa Fluor 488 (Life Technologies, Carlsbad, CA, USA), which selectively binds to actin filaments. Phalloidin, a mycotoxin from the phallotoxin group produced by *Amanita phalloides* mushrooms, specifically targets actin [56]. Cells were first fixed with 3.7% paraformaldehyde for 10 min, washed with phosphate-buffered saline (PBS) at pH 7.4, and then permeabilized using Triton X-100. Phalloidin staining was applied at a 1:200 dilution for 30 min. Fluorescence was detected using excitation/emission wavelengths of 495/518 nm on a TSi Nikon fluorescence microscope (Nikon, Grand River Road Brighton, MI 48116, USA). Additionally, F-actin labeling was analyzed through confocal microscopy with an LSM 510 META Laser Scanning Confocal Microscope.

### 5.3. Mitotracker

To analyze mitochondrial localization, cells were stained with 250 nM Mitotracker Red CMXRos dye (Thermo Fisher Scientific-M7512, Waltham, MA, USA) in culture medium at 37 °C for 45 min, following the manufacturer’s instructions. The analysis was performed using a TSi Nikon fluorescence microscope (Nikon, Grand River Road Brighton, MI 48116, USA), with excitation and emission wavelengths of 579/599 nm, respectively.

#### 5.3.1. Pit Assay

For the pit assay, we used 96-well plates coated with an inorganic crystalline calcium phosphate substrate. Each well received 0.2 mL of osteoclast differentiation medium and was seeded with 6 × 10^5^ hPBMCs. The cells were incubated in a humidified atmosphere with 5% CO_2_ at 37 °C. On day 15, osteoclast resorption activity was quantified by analyzing the mineralized surface. The medium was aspirated, and 100 μL of 10% bleaching solution was added, followed by a 15 min incubation at room temperature. The wells were washed three times with distilled water and then dried for 3–5 h. The plates were subsequently counterstained with the Von Kossa staining kit (Merck KGaA, Darmstadt, Germany) to visualize the substrate. Images of the entire well were captured using a Nikon stereomicroscope, and image analysis software (ImageJ^®^, version 1.54) was used to calculate the percentage of resorbed surface, quantifying osteoclast resorption activity.

#### 5.3.2. Cytometric Bead Array (CBA)

On the 15th day of culture, supernatants were collected, aliquoted, and stored at −80 °C for subsequent cytokine quantification. IFN-γ, TNF-α, IL-2, IL-6, IL-17, IL-10, and IL-4 levels were measured using the Cytometric Bead Array (CBA) Human Th1/Th2/Th17 Cytokine Kit (Becton Dickson, Santo Amaro, SP, Brazil), working according to the manufacturer’s instructions. The analysis was performed using BD Accuri C6 flow cytometry.

### 5.4. Establishment of Non-Lethal Concentrations of Bothrops moojeni Venom and Its Fractions for Osteoclast Precursor Viability Assays

The non-lethal concentrations of *Bothrops moojeni* venom and its fractions were determined for osteoclast precursors in a previous study. This earlier work also described the purification process and the source of the venom. Venom was obtained from adult specimens of *Bothrops moojeni* available at the biobank of the Center of Excellence for the Discovery of Molecular Targets (CENTD). *B. moojeni* venom (10.0 mg/mL) was also fractionated using a 10 kDa cutting membrane, resulting in low- and high-molecular-mass fractions. The established concentrations—5 µg/mL for both the crude venom and HMW fraction, and 1 µg/mL for the LMW fraction—were confirmed so as to not induce significant cell death in osteoclast precursors. These concentrations were chosen to allow for functional assays while preserving cell viability, enabling a detailed investigation of the venom’s impact on osteoclast differentiation and activity [57].

#### 5.4.1. Experimental Setup and Data Generation

The data were obtained with three technical replicates for each study group. The *positive control* consisted of hPBMCs, induced to for OCs, with the maintenance of the culture medium and differentiators without additional treatment. The *negative control* involved hPBMCs cultured in a medium lacking differentiators for 15 days. The study groups included treatment with crude *Bothrops moojeni* venom (5 µg/mL), a high-molecular-weight fraction (5 µg/mL), and a low-molecular-weight fraction (1 µg/mL). The crude venom and its fractions were added on day 4, 7, and 14 of OC differentiation. Treatments were maintained at their respective concentrations, with 50% medium exchange on days 6, 10, and 14 to account for evaporation.

#### 5.4.2. Sample Preparation

Supernatants were collected in sterile Eppendorf tubes with methanol (LiChrosolv^®^)(Merck Millipore, Burlington, MA, USA) at a 1:1 ratio and kept at 4 °C. The samples were centrifuged twice at 10,000× *g* for 5 min each, and the supernatants were discarded. The remaining protein pellets were dried at 30 °C until all methanol had evaporated. The pellets were resuspended in 20 µL of deionized water. Protein concentration was measured using a NanoDrop spectrophotometer (Thermo Fisher Scientific, Waltham, MA, USA) and a BSA curve (Merck Millipore, Burlington, MA, USA). For protein reduction, 20 µL of 50 mM ammonium bicarbonate and 3 µL of 100 mM dithiothreitol (DTT) were added, followed by incubation at 60 °C for 30 min. Iodoacetamide (IAA, 200 mM) was then added, and samples were incubated at room temperature for 30 min in the dark. Trypsin digestion was performed by adding 1 µg of trypsin per 100 µg of protein, with samples incubated at 30 °C for 16 h. The reaction was halted with 10 µL of 5% trifluoroacetic acid (TFA), and samples were dried using a SpeedVac (Thermo Fisher Scientific, Waltham, MA, USA) and stored at room temperature.

#### 5.4.3. Chromatography

The dried samples were reconstituted in 20 µL of ultrapure water and dissolved in 0.1% formic acid (solvent A). The samples were injected into a C18 reverse-phase column (Supelco) with a linear gradient elution starting from 5% to 40% of solvent B (90% acetonitrile) over 66 min at a flow rate of 0.2 mL/min. Eluted components were detected using a mass spectrometer Shimadzu PDA detector at 200–500 nm (Shimadzu, Nakagyo-ku, Kyoto, Japan). A blank run was performed between samples to ensure column cleanliness.

#### 5.4.4. Mass Spectrometry Analysis

Mass spectrometry was conducted using a Shimadzu IT-ToF mass spectrometer (Shimadzu, Nakagyo-ku, Kyoto, Japan) in positive ion mode. The instrument was set to an interface voltage of 4.5 kV, a detector voltage of 1.76 kV, and an interface temperature of 200 °C. Data were acquired across an m/z ratio of 50 to 2000. Collision-induced dissociation (CID) was employed for MS/MS spectra generation, with argon as the collision gas, enabling peptide sequencing and post-translational modification analysis.

#### 5.4.5. Protein Identification

Protein identification was performed using PEAKS Studio 7.0 software with the InChorus multi-algorithm tool, integrating PEAKS and MASCOT algorithms. Protein sequences were compared against public databases, including those of *Homo sapiens* and *Squamata*, enabling broad-spectrum analysis.

#### 5.4.6. Functional Enrichment and Pathway Analysis

Functional enrichment analysis was carried out using FunRich software version 3.1.4 to categorize identified proteins via molecular function, biological processes, and associated pathways. Additionally, Enrichr software Version 3.4 was employed to analyze osteoclast data on mineralized and plastic plates using 21 and 22 databases, respectively. A *p*-adjusted value of less than 0.005 was used for data refinement, and graphs were generated to display consistent pathway and gene enrichment results.

## Figures and Tables

**Table 1:** This table summarizes the pathways and their corresponding proteins that showed significant enrichment in control OCs based on the adjusted *p*-values < 0.05 from the analysis.

Pathway Description	Database	Adjusted *p*-Value	Gene/Complex Involved
Complement cascade	Bioplanet	0.00360	IGHG3; IGHG4; IGHG1; IGHG2
ASCC1	Bioplex	0.00834	IGHG1; IGHG2
ZNF354C	Bioplex	0.00834	IGHG1; IGHG2
SUSD3	Bioplex	0.01085	IGHG1; IGHG2
FAM175B	Bioplex	0.01085	IGHG1; IGHG2
GDPD1	Bioplex	0.01596	IGHG1; IGHG2
ZFP41	Bioplex	0.02269	AKAP1; FBN1
PDE4DIP	Bioplex	0.04499	IGHG1; IGHG2
ZSCAN20	Bioplex	0.04499	IGHG1; IGHG2
PRKAR2B	HuMAP	0.02100	AKAP1
LDLRAD4	HuMAP	0.02100	HECW1
PAX9-MSX1 complex	CORUM	0.00991	MSX1
ITK-SLP-76 complex, anti-TCR stimulated	CORUM	0.00991	ITK
AMY-1-S-AKAP84-RII-beta complex	CORUM	0.00991	AKAP1
SLP-76-PLC-gamma-1-ITK complex, alpha-TCR stimulated	CORUM	0.00991	ITK
Sam68-p85 P13K-IRS-1-IR signaling complex	CORUM	0.01055	IRS1
Doramapimod	HMS	0.00441	MK14; MK11
QL-XI-92	HMS	0.03307	MK14; MK11
CG-930	HMS	0.04648	MK14; MK11
ALW-II-38-3	HMS	0.04648	MK14; MK11

**Table 2:** This table summarizes the pathways and their corresponding proteins that showed significant enrichment in OCs treated with *B. moojeni* crude venom based on the adjusted *p*-values < 0.05 from the analysis.

Pathway Description	Database	Adjusted *p*-Value	Gene/Complex Involved
ALL-1 supercomplex	CORUM	0.04903	SYMPK; CHD3
NRD complex (nucleosome remodeling and deacetylation complex)	CORUM	0.04903	CHD3
Polyadenylation complex (CSTF1, CSTF2, CSTF3, SYMPK CPSF1, CPSF2, CPSF3)	CORUM	0.04903	SYMPK
HDAC2-asscociated core complex	CORUM	0.04903	CHD3
HDAC1-associated protein complex	CORUM	0.04903	CHD3
KCNQ1 macromolecular complex	CORUM	0.04903	AKAP9
HDAC1-associated core complex cII	CORUM	0.04903	CHD3

## Data Availability

The original contributions presented in this study are included in this article and Supplementary Material. Further inquiries can be directed to the corresponding author.

## Supplementary Materials

The following supporting information can be downloaded at: https://www.mdpi.com/article/10.3390/toxins17030141/s1, Table S1. Complete table with the data gene counts HMW fraction. Table S2. Complete table with the data gene counts LMW fraction.
